# Encephaloid Tumor Weighing 36½ Lbs. in a Child Eight and a Half Years of Age—Death—Autopsy

**Published:** 1868-12

**Authors:** C. C. F. Gay

**Affiliations:** Buffalo


BUFFALO

### Medical and Surgical Journal.

VOL. VIII. DECEMBER, 1868. No. 5.

### Original Communications.

ART. I.—*Encephaloid Tumor weighing* 36½ *lbs. in a child eight and a half years of age—Death—Autopsy—Reported by* DR. C. C. F. GAY, *Buffalo.*

Philip Klein was born July, 1860, of healthy parents; he enjoyed good health until he had reached the age of two and a half years. The parents state that at this age the child became greatly frightened by the entrance of a negro into the house, who came for no other purpose than to inquire for work; that immediately after the fright the child voided urine and a few drops of blood; later blood passed in larger quantities, and that a quart was passed in one night, but this no doubt is an exaggerated statement.

Dr. Hauenstein was called, to treat the case, and gave it as his opinion that the blood proceeded from the kidney, a diagnosis which I concurred in when visiting the patient some weeks later along with the doctor. The child continued to discharge blood at intervals for two years and a half, but the greater portion of this time he would feel and act quite well and play about the house. At about this time a swelling was observed in the left lumbar region, which was observed to increase in size very rapidly until soon the whole abdomen became much distended and hard and unyielding to the touch. The child was occasionally visited, more from curiosity than with the hope of rendering medical aid, from this time until some months before death, which occurred on December 1st, 1868, after an illness of six years.

I condense the symptoms and condition of the little patient as given me by the mother during the last two years of illness. The child was able to walk until the first of last August, appetite good, and could "eat more than all the rest of the family," "wanted something to eat every half hour," always thirsty, and drank a great deal of water. The last year did not sleep well, a frequent desire to urinate would awake him, and he had to be got up to relieve this desire; sometimes would pass a large quantity of water, then again but little, and again would go two days without passing any. Did not suffer much pain from the tumor, but would have pain lasting about one week, and then go for a long time without pain. Had diarrhœa for two weeks just previous to death, lasting until four days before death. The bowels during the entire sickness, with this exception, had been regular. The child sat up in his chair the day before his death.

Autopsy performed by Dr. Diehl in the presence of Dr. Hauenstein and myself. Body much emaciated, and, exclusive of the tumor, would not weigh more than forty pounds. I regret that the body was not weighed. Circumference of abdomen forty-three inches; thoracic viscera not examined. On laying open the abdomen the tumor was found to occupy its entire extent; the transverse and descending colon traversed the anterior surface of the tumor; the remaining intestines occupied a position posteriorly to the tumor. The tumor was quite firmly attached in all its parts, but most firmly to the left lumbar region; it was removed nearly entire, and weighed 36½ lbs.; it was lobulated and contained pus depots in some of its parts; portions were hard and unyielding, while other portions were somewhat soft, varying in color, containing blood vessels immense in size, and also containing cysts. Search was made for the left kidney, but it was thought to have been obliterated, but something was found afterwards attached to the tumor supposed to have been the rudiment of the organ; right kidney larger than a normal adult kidney, and weighed six ounces; intestines and bladder normal. To make room within the abdominal cavity for this immense tumor the thorax inferiorly became expanded beyond the average size of the adult.

I have reported this case for the reason, first, that I believe this abdominal tumor to be the largest growth in a child eight and a half years of which there is any record; and secondly, I have reported it on account of the diagnostic value of the symptoms. This is certainly a novel and unique case; fright, to which the parents attributed the illness of their child, was doubtless but a mere coincidence, and had no relation whatever with the causation of the disease. That life could be sustained in a child of this tender age for a period of years, embarrassed by this immense growth, which never even interfered by its pressure upon the intestines, with the regular performance of their function, is most remarkable.

By reference to "Tanner's Index of Diseases," it will be seen that this morbid growth would properly be placed under the head of what he denominates "Renal Cancer," of which hæmaturia is a prominent symptom.

